# The Seventh Annual Session of the American Dental Association

**Published:** 1867-09

**Authors:** 


### *The Seventh Annual Session of the American Dental Association.*

In accordance with the published announcement, this body assembled in Hopkin's Music Hall, Cincinnati, Ohio, on Tuesday July 30th. The meeting was called to order by Dr. Fitch, the President, and after prayer by the Rev. H. D. Moore, an address of welcome was delivered by Dr. James Taylor, the substance of which is as follows: "*Mr. President and Gentlemen of the American Dental Association.*—The very pleasant duty of welcoming you to the Queen City of the West has been assigned to me. We congregate in the same hall in which the Medical Association so recently held their deliberations. "It a matter of no surprise to us, and yet of some congratulation that the subject of medical specialities has assumed with this conservative body some importance. It depends very much on the dentist, the oculist and the *speciality* of general operative surgery, what shall be the status and true progress of medical science in the United States. "In looking back over the rapid progress of dental science for the last thirty years, we feel assured that there is no danger of our being absorbed by the mother of us all, but that the great law of nature will hold good here as well as elsewhere, and that youth, with a good germ life, will soon outgrow, or, at least, overtake maturity." "In looking over the curriculum of our Dental Colleges, we are rather disposed to think we shall soon absorb or lay under contribution every department of medical science. We love, venerate and cherish the time honored profession of medicine. It has embraced for ages a vast array of talent. The learned, the great and good, are from year to year adding fresh lustre to her renown. A more noble, self-sacrificing class of men can nowhere be found; and the more we emulate these noble qualities, and the more of her true science we obtain, the higher we shall place the standard of our speciality.

"This Association is an organ of our professional body, whose function is the developement of dental science. Let us nourish it with so rich a diet that the elementary organs shall rear a structure enduring as time, built on the solid foundations of immutable truth. Having this hope and looking to this end, we now, in behalf of the Mississippi Valley Association, the Ohio Dental College Association, the Cincinnati Association, and such other Societies as rejoice in your presence, bid you a hearty and cordial welcome. We would, Mr. President, through you extend the right hand of fellowship to all who rejoice in the prosperity of our profession, and who may visit our city on this occasion." A committee appointed for the purpose reported the following daily order of business. Clinics from 8 to 9 o'clock, A. M. Morning session from 9 to 12½. P. M. Report of Committee on Instruments and Appliances, from 9 to 9½, A. M. Afternoon session, from 2½ to 6 P. M. Evening session, from 8 to 10 P. M.

Objection was made to the admission of the delegates from the St. Louis Dental College, and the matter was referred to a committee appointed for the purpose.

*Afternoon Session.*—After the reading of the minutes, a motion was made to appoint a committee to report upon the relative status of the dental profession with the Goodyear Rubber Company. The President Dr. Fitch, made some remarks deprecating the introduction of this matter into the proceedings of the Association, since the Association as a body, had nothing to do with that company. The resolution was then laid upon the table, but subsequently Dr. Lawrence of Massachusetts, offered the following, which was adopted: *Resolved,* That this Association does hereby reconsider its action at our last meeting, in regard to the Vulcanite Company, and totally ignore the whole subject. Dr. Watt offered the following: *Resolved*, That members of the Medical Profession of this city, and any who may be sojourning here, be invited to meet with this Association. Amendments were offered and accepted extending the same courtesy to dentists and ministers. Objection was made to this resolution on the ground that no such invitation had been extended by the Medical Convention to the Dentists. The resolution was however adopted. An election was then held, by ballot, which resulted in the choice of the following: President—A. Lawrence. First Vice-President—P. G. C. Hunt. Second Vice-President—A. S. Talbert. Corresponding Secretary—C. R. Butter. Recording Secretary—J. Taft. Treasurer—W. H. Goddard. A committee conducted the President-elect to the chair, who thanked the Association for the honor conferred upon him; expressed his regrets that he could not bring greater executive and other ability to the post than he did, and asked the co-operation of members in maintaining order and the prompt transaction of business.

Dr. Fitch, the retiring President then read an address, briefly reviewing the transactions of the Association during his administration, after which he discussed the nature of *nerve force*, especially in its abnormal state, affecting the practice of dentistry. He enjoined upon his hearers the importance of establishing and maintaining entire compatibility of feeling between them and their patients, in order to success and happy usefulness. An invitation was received from the dentists of Cincinnati and vicinity, to an entertainment on Friday evening August 2nd. Dr. Atkinson remarked that if the entertainment had not already been contracted for and would have been paid for, he would be in favor of rejecting the invitation. The invitation was accepted.

*Evening Session.*—Dr. L. D. Shepherd of Mass. from the Committee on publication of the Transactions of the Association, reported that the volume was nearly completed and would make a fine octavo book of 450 pages. Dr. Shepherd also presented the report of the Treasurer, Dr. W. W. Sheffield, which showed that the receipts and expenditures amounted to $1038.27. Dr. Lawrence read a report on Dental Chemistry, urging upon Dentists the importance of such a knowledge of Chemistry as would enable them to teach their patients what to eat, when and how, so as to preserve their teeth. He insisted that " we sift from our cereals the best portions of the grain and eat the worst."

### *Wednesday's Proceedings.*

*Morning Session.*—On motion a committee was appointed to estimate the financial necessities required by the Association at the present session, which committee reported the liabilities to be $460. A motion was made to increase the annual fees of members to $5, which was at first laid upon the table, but was subsequently adopted, and an additional assessment of $2 made upon each member. A committee was also appointed to report on the action necessary to be taken for the publication of the transactions of the Association; also a committee on Dental Therapeutics. On motion of Dr. Taft, certain amendments to the Constitution, proposed last year, were taken up, one of which was a Code of Ethics, which was discussed at some length, the main question being whether the Association had the right, and whether it was advisable to require local societies to accept and observe such a Code of Ethics. The matter after much discussion, which occupied the greater part of the morning session, was finally laid upon the table.

*Afternoon Session.*—The Association met in Mozart Hall, at 2½ o'clock, having vacated Hopkin's Hall on account of the noise in the street interfering with the business. The committee appointed to consider what disposition should be made of the transactions of this session reported that the publishers of several of the Dental Journals had generously offered to publish all the discussions and written papers presented to the Association free of charge in their respective journals and they recommended that their offer be accepted. This offer was, however not accepted, when Dr. Taft offered to publish the transactions in a separate volume, and look to the sale of the same for his compensation; this also was refused, and on motion of Dr. Spalding the report was laid on the table with the understanding that the Association would publish the transactions as heretofore, at their own expense, in a separate volume. Dr. Kennecott from the Committee on Credentials, reported that they had had the subject of receiving the delegates from the St. Louis Dental College under consideration, and submitted the following: That the St. Louis Dental College has no existence except under a technicality of a loose and dangerous statute of the State of Missouri; that it has never attempted to fulfil the spirit of this bad law by instituting lectures, clinics or any other mode of dental science; that the degrees or diplomas of this so-called College are null and void in law, and ought to be ignored and repudiated by all regularly constituted colleges and associations; that the scheme of its birth was conceived with very discreditable motives, and its culminating act, the sending of delegates to this Association, merits our strongest condemnation and rebuke; and your Committee, believe that the recognition of this so-called Dental College by receiving its delegates, would be setting a dangerous precedent that would lower the standard of dental education, and puts quacks and mountebanks in places of trust and honor in the profession. The report was adopted. Dr. Kennicott offered the following, which, after some discussion, was adopted. WHEREAS, It is a cherished object of this Association to elevate the standard of dental education and wipe out from the escutcheon of our noble profession the stains of quackery; therefore *Resolved*, That we will not countenance any act of any Dental College that shortens the road to the honorable title of Doctor of Dental Surgery by lowering the standard of professional excellence.

Dr. Rehwinkel then moved to take from the table the amendment to the constitution, embodying a code of ethics. After some discussion the motion was lost.

The consideration of the report of the Committee on Dental Chemistry was then announced as the order of the day. At the close of the discussion on Dental Chemistry, Dr. Atkinson made a report on Dental Pathology and surgery, which was peculiar in its character, Dr. A. contending that the presence of periosteum was not necessary for the reproduction of bone. Considerable discussion followed the reading of Dr. Atkinson's report.

*Evening Session.*—The discussion on dental pathology and surgery was resumed, during which cases were mentioned where teeth had been replaced that had been knocked out by violence, to establish the theory that the vital connection between the teeth and the organism of the socket might be restored. This subject occupied the remainder of the evening session.

### *Thursday's Proceedings.*

*Morning Session.*—After the reading of the minutes Dr. Taft read a report from the Committee on suggestions to Dentists, which was approved. The report relates to office students and is embodied in the following form, designed to be sent to Dentists:

The undersigned, the committee appointed according to the provisions of the above resolutions, are fully persuaded that the time has now arrived when every dental practitioner in the country can and should lend his aid in elevating the status of the profession, to the end, that those who are soon to fill our places may be prepared, in a greater degree, to fulfill the reasonable expectations of the public, and hold dentistry in its proper rank among the learned professions.

Our Dental Colleges have done much, and will doubtless do more, but there is a work for the private instructor to accomplish, that students may be better qualified to enter such collegiate institutions, and graduate with credit to themselves and honor to the profession. It is not only essential that Dental Colleges exist, but they should be furnished with properly qualified pupils to insure that success and usefulness contemplated in their foundation.

Relying, then, upon the generous co-operation of our professional brethren, we respectfully submit the following "suggestions" as a basis in "accepting dental students:"

1st. He must possess a good moral character and at least a good English Education.

2d, He must be required to apply himself diligently for three years, including two full courses of lectures in some Dental College, to the following studies, viz:

First Year—Anatomy, Histology and Physiology.

Second Year—Pathology, Chemistry, Metallurgy and Mechanical Dentistry.

Third Year—Operative Dentistry, Special Pathology, Dental Medicine and Microscopy.

We further suggest that the instructor examine his pupil in his studies at least twice every week, and as much oftener as may be convenient—not, however, including his lecture terms—and should the students, after sufficient trial, fail to exhibit the necessary talent for our specialty, he should be kindly apprised of the fact, and advised to seek other fields of usefulness.

Practitioners favoring the foregoing are respectfully requested to date and sign the accompanying paper and forward to the Committee. A. Lawrence, Lowell, Mass.; C. P. Fitch, New York City; J. Taft, Cincinnati, Ohio.

The question where the next place of meeting should be held was taken up, and on the second ballot Niagara Falls was declared the choice of the Association. Dr. Allen of New York then read a paper on the Physical History of various nations of the Earth, with special reference to their teeth. This report was very interesting and instructive, embracing a cursory review of the character and condition of the teeth of various nations. The points Dr. Allen endeavoring to demonstrate were: 1st, That the teeth of the people of this country are worse than those of other nations of the world; and, 2nd, To show the cause of such bad teeth in America.

Some nations keep their teeth till old age, and lose them as rarely as they do their arms or limbs. In this country, twenty millions of teeth are lost annually from decay. In examining the cause of this loss, it may be stated that the inhabitants of Europe who discard the mineral elements of food, seldom loose their teeth by decay. The Albanians live on milk, cheese, eggs, and boiled maize. Their teeth are fine, and remain good to old age.

The mountain tribes of Asia, especialy the Tartars, have strong, white teeth. The teeth of the Arabs are white and regular. They eat seldom, and never of animal food.

Dr. Allen next mentioned the inhabitants of the East Indian Islands, Zealanders, &c., as having good teeth, which lasted to old age

The American Indians, as a general thing, had good teeth, which are large, never decayed, although worn, as in old age, by use.

The Chilians, Californians, and inhabitants near the bay, all have fine, well set teeth. Their diet is farinaceous. The inhabitants of Peru and Patagonia have beautiful teeth, even in old age.

With regard to the bad teeth of the Americans, it would be said by the candid dentist that they were set in narrow, contracted jaws, and were badly decayed.

Humboldt said of the Camas, that they had fine teeth, like all people who lead a simple life. No nation that changed their food from the condition in which nature furnished it, had good teeth.

Plenty of exercise and fresh air was another cause of the good teeth in the swarthy races.

We have attempted to improve our bread by bolting the flour, but thereby destroyed the mineral elements which go to form the teeth. There are 13.868 mills in the United States: 27,000 men are required to work them, and it requires nearly $9,000,000 annually to change the constituents of our food, and the result undoubtedly is one of the most prominent causes of the destruction of Americans' teeth. Lime is to be found in the outer portion of the grain; and it is needed for the teeth, yet we reject it. It is for the profession of dentistry to do good by diffusing such knowledge as comes to it through experience and research to prevent the evil of premature decay and loss of the teeth.

On motion, Dr. Allen's paper was referred to the Committee on Publication.

The subject of gold fillings was then introduced by Dr. J. F. Flagg of the Philadelphia College who spoke in the highest terms of Morgan's "Plastic gold," Dr. Flagg claimed for this material that a like amount of skill can produce an equal result with much greater ease than with foil; that it contains nothing injurious to tooth substance; that it is purer than gold foil, as has been proved by assays made at the mint; also, that he found in addition to these qualities, excessive ease of manipulation, rapidity of filling, (so much so that a filling may be introduced in a less time than two minutes,) peculiar adaptability to walls of cavity ease of finish requisite solidity, beauty of color, absence of crumbling, thoroughness of compacting, and freedom from waste of material. The assertion made by Dr. Flagg that a filling of this form of gold can be introduced in considerably less time than two minutes met with considerable opposition, many of the members of the Association expressing themselves unfavorably to the theory of the work being properly accomplished in the short space of time—two minutes—as asserted by Dr. Flagg.

*Afternoon Session.*—Dr. Atkinson moved that the Convention hear Dr. Cutler's paper on microscopy at 4 o'clock.

The report of the Committee on Operative Dentistry was read by Dr. Butler, also a paper under the same head by Dr. Palmer, in which he advocated the use of three wedges for separating teeth.

Dr. Russell of Nashville, made some statements on the theory of filling teeth, interlarding his observations with humorous anecdotes and illustrations. In the course of his remarks he stated that those Professors who mostly charged the highest price, generally made the least money, from the fact that they took longer time to do their work effectually. He instanced the case of the celebrated Dr. Badger, who had informed him that the highest amount he had received for a day's work was in New Orleans, when he got $55. The specified time having arrived, Dr Cutler of Mississippi read a lengthy and scientific essay upon the "microscopy of the nerves of the Dental Pulp." His views he claimed were supported by practical experimental observation. One proposition was that a molar tooth did not contain less than one hundred thousand nerves.

*Evening Session.*—A sharp discussion ensued upon the paper of Dr. Cutler, on the "microscopy of the nerves of the Dental pulp" carried on mainly by Drs. Atkinson, Cutler, Judd, Wetherby, Spalding, and Chase. Some of these gentlemen accusing Dr. Cutler of supporting a superannuated worn-out theory. Dr. Cutler in reply remarked that he did not claim there were no errors in his report, but he wished them sifted from the truths, and not as you would sift out the nutriments from a barrel of flour. He said he had specimens to sustain his proposition, that the tubuli of dentine contained nerve matter, and that he had expended at one period alone, three months of earnest days and almost sleepless nights, in his investigations, and yet he might say that even now he had hardly commenced the anatomy of that little nerve. The question whether the tubuli contained nerve matter or fluid, occupied the entire evening session, and the discussion ended without a settlement of matter in dispute.

### *Friday's Proceedings.*

*Morning Session.*—Considerable time was occupied in the discussion of a motion to pay the Secretary $50 per annum, which was lost. The report of the committee on instruments and appliances was received. Dr. French offered a resolution that the sum of $5.000 be offered as a prize to any chemist, or other experimenter, who shall invent, for the use of the dentist, a perfectly plastic material, that shall be in every respect be equal to gold as a filling for decayed teeth, and shall more nearly approximate the teeth in point of color.

After some discussion this resolution was laid on the table.

Dr. Spalding offered the following which was adopted: *Resolved*, that the Treasurer be instructed to furnish annually, to the chairman of the Publication Committee, a list of all who have paid their annual dues, for his guidance in the distribution of the annual volume of transactions. The subject of Operative Dentistry was then taken up, and on motion, Dr. Barnum illustrated his method of applying the Rubber Dam.

*Afternoon Session.*—Dr. Bogue offered the following resolution: *Resolved*, that it is the duty of each member of the Standing Committee to make an individual report, so far as such report can be made, and in case of inability to be present at the meeting, to forward it to the Recording Secretary, from whom it can be obtained by the chairman of each committee, respectively, at the time of assembling of this Association. Adopted.

The Nominating Committee then made their report of the Standing Committees for next year, and the Committee on Publication recommended the appointment of a special committee of three, to receive and take care of articles presented for examination and exhibition at the annual meeting, which was adopted, and Drs. L. D. Shepherd, E. A. Bogue, and Walker appointed as said committee.

The greater part of the afternoon was occupied in discussing the report on mechanical dentistry, during which several interesting cases were reported verbally. Dr. Herriott related that he had made out of English rubber, an artificial nose and upper lip for a soldier who had had both, together with his upper teeth and part of the lower ones, carried away by a piece of shell at Mission Ridge. Dr. Herriott presented photographs of the patient before and after the operation, showing a marked improvement in appearance, and stated that the speech was improved fully as much as the face. Drs. Osmond and Osgood presented cases of cleft palate together with descriptions of the apparatus they applied for its remedy. In the evening an entertainment was given to the association in Hopkin's Hall.

### *Saturday's Proceedings.*

The association met at 9 o'clock, A. M., when the Committee on Dental Physiology reported. Dr. H. S. Chase read an interesting paper on this subject, and also one in the "Absorption of Deciduous Teeth." The report on microscopy was received and referred to the Committee on Publication. A committee was appointed to draft resolutions for next year, expressive of the sense of the Association on deceased members. The Committee of Investigation on decay was continued until next year. The business of the Association having been concluded, the President made a few closing remarks, and adjourned the convention, to meet at Niagara Falls on the 30th of July next.

